# Microneedle delivery system with rapid dissolution and sustained release of bleomycin for the treatment of hemangiomas

**DOI:** 10.1186/s12951-024-02557-7

**Published:** 2024-06-25

**Authors:** Bin Sun, Tikai Zhang, Hongrui Chen, Wei Gao, Jingwei Zhou, Yuxi Chen, Wang Ding, Xiaofan Yin, Jie Ren, Chen Hua, Xiaoxi Lin

**Affiliations:** 1 Department of Plastic and Reconstructive Surgery, Shanghai Ninth People’s Hospital, Shanghai Jiao Tong University School of Medicine, Shanghai, 200011 China; 2 Institute of Nano and Biopolymeric Materials, School of Materials Science and Engineering, Tongji University, Shanghai, 201804 China; 3 Department of Orthopaedic Surgery, Minhang Hospital, Fudan University, 170 Xin Song Road, Shanghai, 201100 China

**Keywords:** Hemangioma, Bleomycin, Microneedle, Polylactic acid, Microsphere

## Abstract

Hemangioma of infancy is the most common vascular tumor during infancy and childhood. Despite the proven efficacy of propranolol treatment, certain patients still encounter resistance or face recurrence. The need for frequent daily medication also poses challenges to patient adherence. Bleomycin (BLM) has demonstrated effectiveness against vascular anomalies, yet its use is limited by dose-related complications. Addressing this, this study proposes a novel approach for treating hemangiomas using BLM-loaded hyaluronic acid (HA)-based microneedle (MN) patches. BLM is encapsulated during the synthesis of polylactic acid (PLA) microspheres (MPs). The successful preparation of PLA MPs and MN patches is confirmed through scanning electron microscopy (SEM) images. The HA microneedles dissolve rapidly upon skin insertion, releasing BLM@PLA MPs. These MPs gradually degrade within 28 days, providing a sustained release of BLM. Comprehensive safety assessments, including cell viability, hemolysis ratio, and intradermal reactions in rabbits, validate the safety of MN patches. The BLM@PLA-MNs exhibit an effective inhibitory efficiency against hemangioma formation in a murine hemangioma model. Of significant importance, RNA-seq analysis reveals that BLM@PLA-MNs exert their inhibitory effect on hemangiomas by regulating the P53 pathway. In summary, BLM@PLA-MNs emerge as a promising clinical candidate for the effective treatment of hemangiomas.

**Graphical Abstract:**

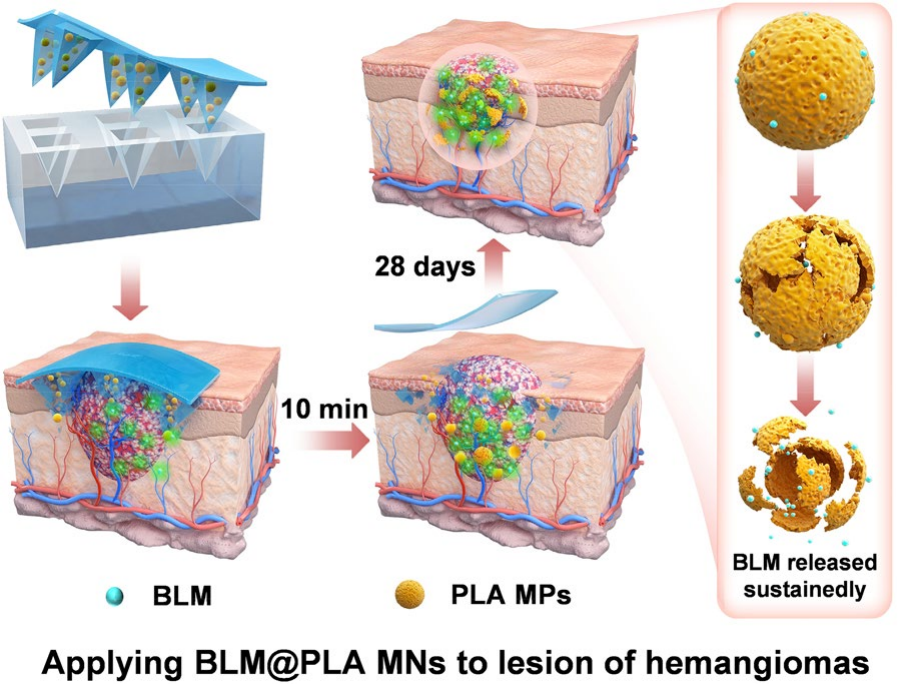

**Supplementary Information:**

The online version contains supplementary material available at 10.1186/s12951-024-02557-7.

## Introduction

Hemangioma of infancy is the most prevalent vascular tumor and the most common benign neoplasm during infancy and childhood, with historical incidence rates ranging from 2 to 10% and an overall prevalence of 4.5% [1]. Hemangiomas are categorized as infantile or congenital. Infantile hemangiomas (IHs) are characterized by rapid postnatal proliferation lasting for 3–6 months, followed by slow regression over years [2]. However, about 10–15% of larger hemangiomas or those in specific locations, such as the head, neck, perineal, and anal regions, may lead to complications like ulceration, disfigurement, or obstruction during the proliferative stage [3]. Unlike IHs, which typically enlarge after birth and then involute, congenital hemangiomas (CHs) proliferate in utero and are fully developed at birth. Three major subtypes of CHs are defined: rapidly involuting congenital hemangioma (RICH) [4], noninvoluting congenital hemangioma (NICH) [5] and partially involuting congenital hemangioma (PICH) [6]. RICH undergoes complete involution within the initial 6 to 14 months whereas NICH expands in proportion to the child's growth without regression.And PICH regresses incompletely and then stabilizes. CHs may exhibit persistent ulceration, hemodynamic instability, and thrombocytopenia [7]. Hence, besides hemangiomas that naturally regress, active treatments are necessary for hemangiomas that do not regress and are accompanied by complications.

Currently, three primary treatment methods exist for hemangiomas: medication, laser therapy, and surgery [8]. Laser therapy and surgery, although effective, come with a relatively high cost and may not be suitable for every patient. Consequently, non-invasive, cost-effective, and convenient oral medications have become the preferred choice since 2008. Propranolol, a nonselective beta-blocker, has emerged as the first-line therapy for complicated infantile hemangiomas [9]. Notably, Hemangeol (propranolol hydrochloride oral solution) stands out as the only FDA-approved drug with proven safety and efficacy for treating infantile hemangiomas [10, 11]. However, potential adverse effects of Hemangeol include sleep disorders and aggravated respiratory tract infections [12]. The requirement for twice-daily oral administration may also reduce patient adherence. Additionally, 0.9% of reported cases were resistant to propranolol, and 18% experienced recurrence after ceasing oral drugs [13]. Meanwhile, timolol maleate has also been widely studied for topical application for IH treatment due to the commercial availability of timolol ophthalmic drops. For both propranolol and timolol, topical treatment is typically applied multiple times a day, which can reduce adherence to therapy [14, 15].

Bleomycin (BLM), a chemotherapy agent that inhibits DNA synthesis, is gaining increased prominence in sclerotherapy for patients with vascular anomalies [16]. Our team has reported the effectiveness of intralesional bleomycin injection in treating hemangiomas resistant to propranolol [17]. However, the repeated use of BLM is associated with potential dose-related complications, such as pneumonitis and pulmonary fibrosis, significantly limiting its practical application [18, 19]. In contrast, topical dermal application of BLM is considered a more favorable option for treating superficial hemangiomas. This method minimizes undesirable effects compared to oral administration, as it can achieve a high concentration of the drug locally while reducing systemic exposure. Despite these advantages, the transdermal transport efficiency of most drugs is hindered by the stratum corneum, leading to unsatisfactory therapeutic effects in the majority of cases [20, 21]. Consequently, there is an urgent need to develop effective strategies to improve the efficiency of topical drug delivery.

Microneedle (MN) patches, which are topical transdermal drug delivery systems consisting of arrays of micrometer-sized needles and substrates, have seen significant development in recent years [22, 23]. These microneedles create transport pathways through the skin's stratum corneum to deliver various therapeutic molecules [24, 25]. Various materials have been designed for MN patches recently [26]. Non-degradable MN structures generally exhibit excellent mechanical properties, ensuring the structural integrity of MNs during use [27]. However, the release of the active pharmaceutical ingredient (API) from non-degradable MNs can continue for several days or even weeks, making long-term wear inconvenient and aesthetically displeasing [24]. Therefore, rapidly biodegradable (dissolvable) MN patches may be more suitable for treating hemangiomas. Additionally, the customizability of microneedle size, including the patch size and the height of the tips, can meet the specific needs of individual patients in clinical applications.

Hyaluronic acid (HA), a widely used water-soluble biomacromolecule, exhibits excellent biocompatibility [28]. HA-based MNs achieve rapid drug delivery by separating the needles from the base [29, 30] and regulating the amount of drug released by controlling the loading capacity of the drug, leaving no poisonous residue after insertion [31]. However, simple HA-based MN patches dissolve too rapidly upon injection into the skin [32], resulting in swift metabolization and release of the API. This necessitates frequent usage within a short timeframe, elevating the risk of bacterial infection [33]. Thus, extending the drug release time with the shortest use of microneedle patches is a key consideration.

Polylactic acid (PLA), one of the most promising biodegradable polymers, is derived from renewable natural plants such as corn, wheat, rice, and sugar cane [34, 35]. It can be degraded into water and carbon dioxide in vitro or low molecular weight PLA and innocuous lactic acid in vivo. PLA microspheres have been used for the delivery of different drugs, including small molecule drugs, polymers, and proteins. With the increasing demand for eco-friendly polymeric materials in the biomedical field, such as implantable biomaterials and medicine packages, PLA has become one of the most promising alternatives [36, 37].

To develop a more efficient therapy for treating hemangiomas, we propose a simple strategy for synthesizing BLM-loaded HA-based MN patches. BLM was encapsulated during the synthesis of PLA microspheres (MPs). The successful preparation of PLA MPs and MN patches was confirmed through scanning electron microscopy (SEM) images. Furthermore, we investigated the in vitro release profile of BLM from PLA MPs. The safety of the MN patches was comprehensively validated through assessments of cell viability, hemolysis ratio, and irritation and skin sensitization tests on rabbits. Subsequently, we established a murine hemangioma model and demonstrated the inhibitory effect of BLM@PLA-loaded MN patches on hemangiomas by modulating the P53 pathway.

## Materials and methods

### Materials

HA (Mw: 90,000) was purchased from MEILUNE Biomed. PLA, Bleomycin and polyethylene glycol were obtained from Macklin. CHCl_3_ were purchased from Sigma-Aldrich. Deionized water was used throughout all the experiments. Polydimethylsiloxane (PDMS) master molds of MNs (320 μm base diameter, 830 μm height, and 20 × 20 arrays with 650 μm tip-tip spacing) were provided by Taizhou Microchip Pharmaceutical Technology Co., Ltd, China.

### Characterization

The hemoglobin release was recorded by the OD values at 576 nm tested by the Eon microplate spectrophotometers (Bio Tek Instruments, Inc.). Thermogravimetric Analysis (TGA) was performed on TGA 4000 (Perkin Elmer Co., Ltd) with the temperature ranged from 30 to 800 °C under N_2_ at a heating rate of 10 °C min^−1^. The SEM images were examined by ZEISS Gemini 300 fitted with Oxford Xplore EDS detector. The TEM images were examined by JEOL2100F fitted with JED2300 EDS detector. Fourier transform infrared (FT-IR) spectra of the patches were recorded by a Thermo-Scientific Nicolet 6700 FT-IR spectrometer (4000–400 cm^−1^). X-ray Photoelectron Spectroscopy (XPS) measurements were recorded on Thermo-Scientific K-Alpha with Al Kα radiation. UV–visible spectroscopy was tested with SHIMADZU UV-3600i Plus.

### Synthesis of PLA and BLM@PLA MPs

**Fig. 1:**
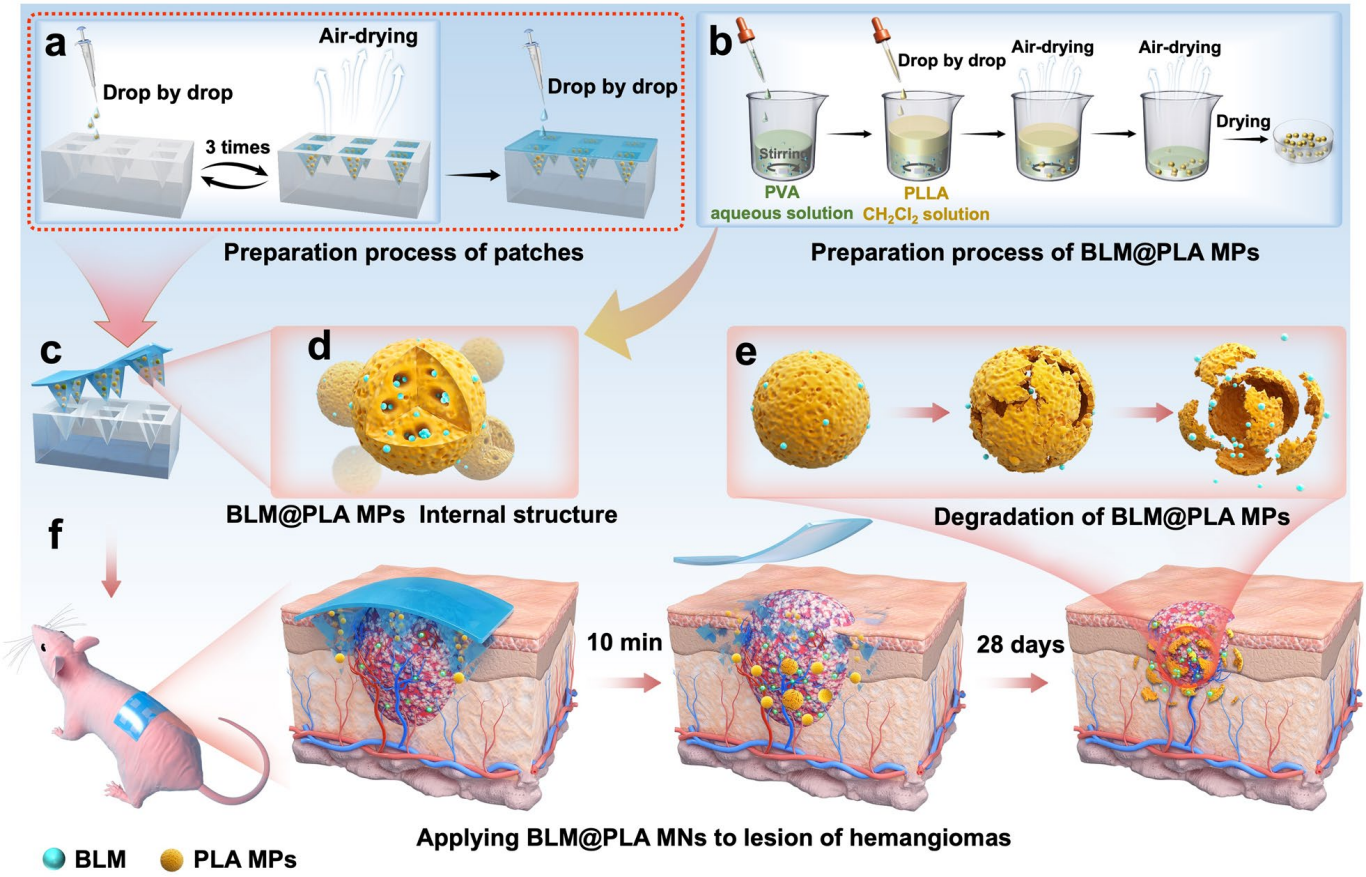
Schematic of BLM@PLA MPs loaded MN patches controlling drug release and treating hemangiomas. **a** The preparation process of MN patches. **b** The fabrication process of BLM@PLA MPs. **c** The illustration of the BLM release process. The right insert presents the internal environment of BLM@PLA MPs. **d** Internal structure diagram of MPs. **e** The degradation process of MPs in subcutaneous tissue. **f** The entire process of applying BLM@PLA-MNs to treat hemangiomas

The PLA MPs were synthesized as the previous work reported [38]. Blank PLA MPs were prepared through emulsion solvent evaporation method as shown in Fig. 1. PLLA (5 g) particles and Polyvinyl Alcohol (PVA) were dissolved in CHCl_3_ (100 mL) and deionized water (100 mL) respectively. The PLLA solution was then added to aqueous PVA solution under magnetic stirring at a rotation speed of 900 rpm. The emulsion was stirred continuously overnight until the CHCl_3_ evaporated completely. Afterwards, the mixed emulsion was filtrated with a double-layer sieve mesh (pore diameters were 7.5 μm and 5 μm respectively) and washed by deionized water to remove the residual PVA. The PLA MPs which remained between the sieve mesh were freeze-dried and collected for further use.

The BLM@PLA MPs were prepared via one-step synthesis by mixing BLM (3 mg) to the PVA solution in the first step of synthesizing PLA MPs and followed the similar process with PLA MPs.

### Preparation of HA-based MN patches

A customized PDMS master mold of MNs (320 μm base diameter, 830 μm height, and 20 × 20 arrays with 650 μm tip-tip spacing) was used to fabricate MN patches. The HA aqueous solution (5 wt%, 1 mL) and BLM (30 mg), PLA MPs (30 mg) or BLM@PLA MPs (30 mg) were ultrasonicated for 30 min and subsequently cast into the PDMS mold until just filling up the all cavities. Afterwards, the mold filled with solution was degassed by vacuum oven for 30 min at room temperature, and the mold was air-dried overnight at room temperature for complete drying and the processes repeated for 3 times to fully fill all cavities. And then place the mold in dry environment for further use. Finally, additional 2 mL HA aqueous was cast into the mentioned mold above gradually, and the mold was placed for further air-drying for 24 h, after which the patch was carefully peeled from the mold and stored in a desiccator until use. Specifically, BLM, PLA and BLM@PLA MPs loaded MN patches were denoted as BLM-MNs, PLA-MNs and BLM@PLA-MNs respectively.

### Loading efficiency of BLM in PLA MPs

The loading capacity of BLM could be calculated via thermo-gravimetric analysis curves of PLA and BLM@PLA MPs. Specifically, after heating 5 mg of BLM, PLA and BLM@PLA MPs from 30 to 800 °C respectively (heating rate: 10 °C min ^−1^), the BLM content in BLM@PLA could be accurately calculated with the remaining residue. The detailed formula of drug loading efficiency was presented as follows (A, B and C represent as PLA, BLM@PLA and BLM respectively):

$$\text{BLM loading rate }\left(\% \right)= \frac{{A}_{\text{residual rate}}- {B}_{\text{residual rate}}}{{A}_{\text{residual rate}}- {C}_{\text{residual rate}}} \times 100 \%$$

### In vivo intradermal irritation experiment

All procedures followed were in accordance with the Declaration of Helsinki. All procedures were reviewed and approved by Shanghai Ninth People's Hospital Central Lab IACUC (Permit Number: SYXK (Shanghai) 2016-0016), and all experiments conformed to the relevant regulatory standards. New zealand white rabbit (2.5–3.5 kg) were purchased from the Shanghai JieSiJie Laboratory Animal Co.Ltd and housed according to ISO 10993-2 regulations.

In this experiment, samples(HA-MN/PLA-MN/BLM-MN/BLM@PLA-MN) were extracted using 0.9% saline solution (polar) and sesame oil (Non-polar) at 37 °C, 60 rpm constant temperature shaking incubator for 72 h. Negative control solution was prepared under the same conditions. Eighteen hours before the experiment, thoroughly remove the fur from both sides of the animal's spine on the back. On the day of the experiment, 0.2 mL of the polar extracts prepared with 0.9% saline were injected intradermally at five points on one side of the rabbit's spine. On the other side of the spine, polar solvent control (0.9% saline) solution was injected. The non-polar extract and the non-polar control solution were handled in the same manner. The erythema, edema, and necrosis of injection sites were observed and recorded immediately, at (24 ± 2) h, (48 ± 2) h, and (72 ± 2) h after the injections. The dorsal skin of rabbit was excised for histological examination.

### Cell separation and culture

Three patients diagnosed with NICH were enrolled in the study. Specimens of these individuals were collected through surgical treatment at the Shanghai Ninth People’s Hospital, Shanghai Jiao Tong University School of Medicine (Shanghai, China). The study was approved by the Institutional Medical Ethical Review Board of Shanghai Ninth People’s Hospital. Informed consent was provided for the specimens, according to the Declaration of Helsinki. The clinical diagnosis was confirmed by both the Department of Pathology and the Department of Plastic and Reconstructive Surgery at Shanghai Ninth People’s Hospital. Detailed patient information is provided in Supplementary Table S1.

Vascular endothelial cells were isolated from specimen of congenital hemangioma. The tissue was digested with 0.2% collagenase I (Roche Diagnostics, Indianapolis, IN).The tissue homogenate was filtered through a 70-μm cell strainer (Fisher Scientific, Hampton, NH). Red blood cells (RBCs) were lysed using Red Blood Cell Lysis Buffer (C3702,Beyotime). Then, centrifuge at 300*g* for 10 min, discard the supernatant. Resuspend cells in pre-chilled PBS. Resuspend cells in 100 μL buffer for every 10^7^ cells. Vascular endothelial cell of congenital hemangioma selected using anti-CD31-coated magnetic beads (Miltenyi Biotec, Germany). CD31-selected cells were cultured in Endothelial Cell Growth Medium-2 (C-22011, PromoCell) with 10% fetal bovine serum and 1% antibiotics. All cells were incubated at 37 °C in a carbon dioxide incubator set at 5%.

### In vivo murine model of congenital hemangioma

Six-week-old female nude mice were obtained from Shanghai JieSiJie Laboratory Animal Co.Ltd. The mice were anesthetized by 2% chloral hydrate (0.004 mL/g) and an ABS rodent anesthesia machine (Yuyan Corporation, Shanghai, China). The vascular endothelial cells were (1 × 10^6^) were suspended in 100 μL of 1:1 PBS and Matrigel TM (BD Bioscience) and subcutaneously injected into the right flank of 6-week-old female nude mice (n = 3 per group). Mice were subjected to MN patches treatment starting 2 weeks after cell injection. The Matrigel implants were collected 4 weeks after MN patches treatment. All mice were euthanized after implants collection. These transplants were collected for H&E, immunohistochemistry (IHC) and immunofluorescence (IF). The microvascular density was measured using a previously described technique [39], Microvascular counting was performed within the same field at a magnification of 200× (0.74 mm^2^). Subsequently, the number of microvessels per 1 mm^2^ was calculated and referred to as "microvascular density (counts/mm^2^)."For IHC and IF, five images in the slides were randomly selected from each group and captured under microscopy.

### RNA-based next-generation sequencing (RNA-seq)

Total RNA of vascular endothelial cells of hemangiomas treated with bleomycin or PBS 24 h was purified using RNeasy mini kit (Qiangen, Germany). The preparation of the complementary DNA library and sequencing followed the standard protocol provided by Illumina. For the analysis of differential gene expression, the edgeR package was employed. Genes with an absolute log2 fold change greater than 1 and a q-value less than 0.05 were considered significantly modulated and retained for further analysis. Pathway analysis was performed using Gene Set Enrichment Analysis (v3.0). (SRP472027).

### Statistical analysis

Analyses were performed by GraphPad 7.0 software. Data are presented as mean ± standard error of the mean (S.E.M.). Unpaired two tailed Student’s t-test and one-way ANOVA were utilized as appropriate. No samples, mice or data points were excluded from the analyses. For all analyses, results were considered statistically significant with *p < 0.05, **p < 0.01, ***p < 0.001.

## Results and discussion

### Design and fabrication of BLM@PLA-MNs

In order to meet clinical requirements for treating hemangiomas, we designed and developed a drug delivery system that combines rapid drug administration with slow release. Hyaluronic acid was chosen to prepare the microneedles due to its excellent biocompatibility and water solubility (Fig. 1a). BLM is encapsulated in PLA MPs and delivered subcutaneously via microneedles for drug release (Fig. 1b–e).

The schematic diagram illustrating the effect of hemangioma treatment is shown in Fig. 1f. The internal environment of BLM@PLA MPs is presented in Fig. 1d. After removing the patches from the mold, they are attached to the nude mouse lesion. Following 10 min of subcutaneous insertion, the MN substrate is removed, and the MPs are rapidly released to the lesion along with the tips degrading. Subsequently, BLM is slowly released topically from PLA MPs, facilitating long-term inhibition and treatment of hemangiomas over a 28-day period (Fig. 1e and f). Compared with the direct subcutaneous release of BLM, this strategy can maintain therapeutic effects for a more extended period. Additionally, the rapid separation of MN tips from substrates reduces the negative impact of long-term wearing of microneedle patches on patients' daily lives.

### Synthesis and characterization of PLA and BLM@PLA-MNs

Polylactic acid (PLA) exhibits excellent biocompatibility and degradability. In comparison with HA, its degradation rate is relatively slow, ensuring the long-term subcutaneous release of BLM. Moreover, PLA MPs can be prepared under relatively mild conditions, facilitating drug loading without compromising the bioactivity of BLM. BLM@PLA was prepared using an in-situ drug encapsulation method.

**Fig. 2:**
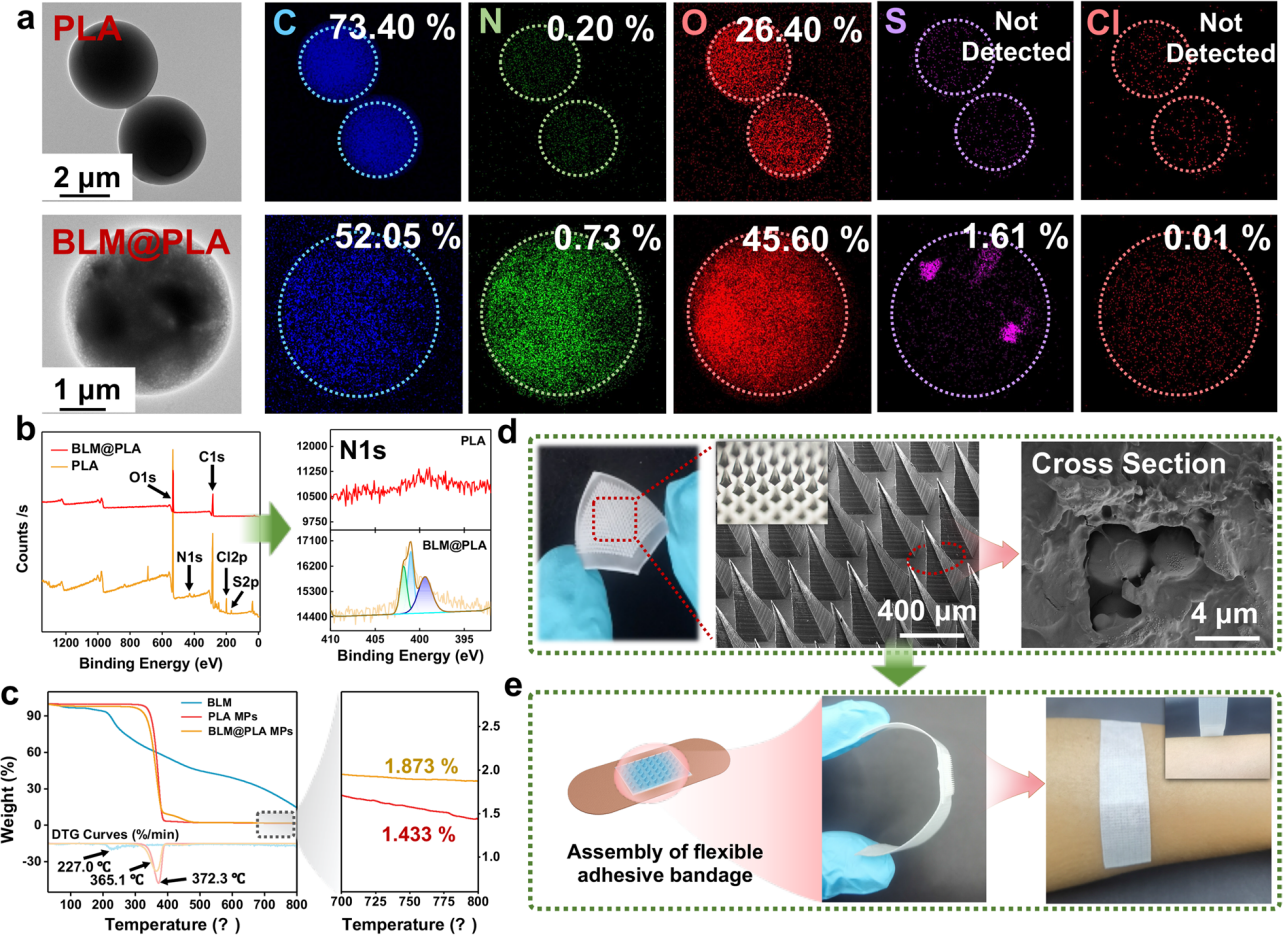
Characterization of PLA MPs and MN patches. **a** TEM images of PLA and BLM@PLA MPs, along with EDS analysis of MPs. **b** XPS spectrogram and high-resolution spectra of N1s of PLA and BLM@PLA. **c** Thermo-Gravimetric Analysis of PLA and BLM@PLA MPs. **d** Optical photography, SEM image, and microscopy image (inserts) of BLM@PLA-MNs. **e** The schematic diagram of the assembled flexible adhesive bandage and its actual wearing

TEM images revealed the morphology, particle sizes, and regular spherical shape of PLA MPs. The shape and size of the MPs were maintained even when BLM was encapsulated within them (Fig. 2a). Chemical analysis conducted via energy dispersive X-ray spectrometry (EDS) showed the presence of C, N, O, S, and Cl elements in both PLA and BLM@PLA (Fig. 2a, Figs. S1 and S2). Compared with PLA MPs, the N element content of BLM@PLA MPs increased dramatically (from 0.20% to 0.73%), suggesting the successful encapsulation of BLM in PLA MPs. Additionally, S and Cl elements were not detected in PLA MPs, while their contents in BLM@PLA were 1.61% and 0.01%, respectively, due to the loading of BLM. It's worth noting that the C and O element content changed to a certain degree after BLM was encapsulated (from 73.40% to 52.05% and 26.40% to 45.60%, respectively). This change can be attributed to the distinct proportions of C and O in BLM and PLA. Similar results were observed in the XPS spectrogram of MPs (Fig. 2b). The peak at 395–405 eV corresponded to the N element. The change in N element content was consistent with the EDS analyses, further confirming the successful encapsulation of BLM. Subsequently, the high-resolution (HR) spectra of N1s were analyzed by differentiating and fitting different peaks at the binding energies of 399.4, 401.0, and 401.8 eV, attributed to nitrogen atoms on the –C–NR2 (R = C/H), nitrogen atoms of heterocyclic amines, and –N–(C=O), respectively. Similarly, the S and Cl elements of MPs increased significantly after loading BLM (Fig. S3).

The loading efficiency of BLM was preliminarily investigated via UV–Vis spectrogram of BLM in an aqueous solution (Fig. S4). The characteristic peak of BLM apparently decreased after encapsulation. The thermal stabilities of BLM and MPs were measured under a nitrogen atmosphere at a heating rate of 10 ℃ min^−1^ (Fig. 2c). The degradative weight loss of BLM occurred at approximately 227.0 ℃, well beyond the temperature range for synthesizing BLM@PLA. While the degradation temperature of BLM@PLA MPs slightly decreased compared to PLA MPs due to BLM decomposition, the residue rates for PLA, BLM@PLA, and BLM were 1.43%, 1.87%, and 14.76%, respectively, with a 0.43% weight loss difference between the two MPs. Thus, the drug loading rate of BLM can be calculated as 3.29 wt % according to the formula mentioned above. These results above indicate the successful synthesis of BLM@PLA-MNs.

### Fabrication and characterization of MN patches

The MN patches were prepared using a two-step method. Optical photography revealed that the synthesized MN patches were colorless and transparent (Fig. 2d). SEM, optical microscopy, and fluorescent microscope images of the patches showed that all needles had a pyramidal shape, with approximately 320 μm sides at the base, 830 μm in height, and a tip-to-tip spacing of about 650 μm (Fig. 2d and insert, Fig. S5). The surfaces of the needles and substrate were uniform and smooth. The cross-section of the tips was proven to be porous via SEM images. To further illustrate the clinical use of MN patches, we assembled a flexible adhesive bandage with the cohesive bandage and MN patches. The practical wearing diagram was exhibited in Fig. 2e. The adhesive bandage was flexible and easy to tear off from the skin surface, demonstrating its potential practical clinical application.

**Fig. 3:**
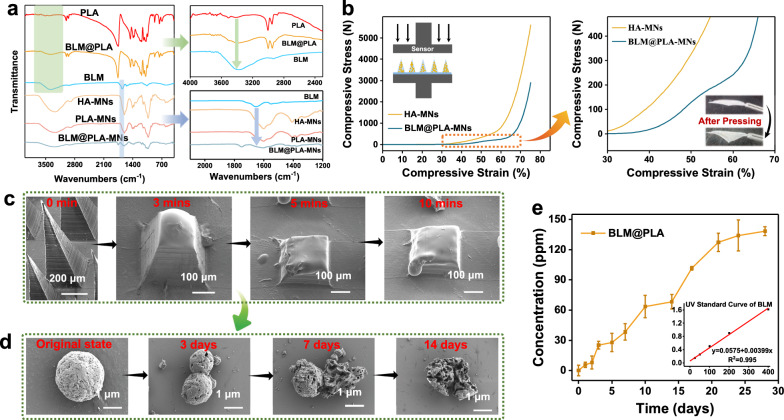
Characterization and degradation of BLM@PLA-MNs. **a** FT-IR spectroscopy of NPs and MN patches. **b** Graphs depicting the mechanical behavior of the patches under compression applied by a vertical force (the insert includes schematics of the experimental setups, and the right side shows amplified curves between 30 and 70% strain). **c** SEM images of MN tips after insertion into fresh rabbit cadaver skin for 10 min. **d** SEM images illustrating the degradable process of BLM@PLA in PBS (pH = 7.4). **e** In vitro transdermal release assays of BLM from MN patches. Bars represent means ± SD (n = 3 independent samples)

The chemical structures of the fabricated MN patches were characterized by FT-IR spectroscopy (Fig. 3a). The absorption peaks of all PLA MPs at 756 cm^−1^ were assigned to the methyl group of PLA. The characteristic peaks of BLM and BLM@PLA at 3310–3350 cm^−1^ were assigned to the secondary amine of BLM. The characteristic peak at 1750–1760 cm^−1^ was attributed to the C=O of PLA. Additionally, the characteristic peak at 1150–950 cm^−1^ was attributed to the monosaccharide of HA. Moreover, the characteristic peaks at around 1650 cm^−1^ were due to the C=O of BLM. These results confirmed the successful fabrication of BLM@PLA-MNs.

### Mechanical strength of BLM@PLA-MNs

To investigate the influence on the mechanical properties of introducing PLA MPs to MN tips, we compared the differences in compressive properties between HA-MNs and BLM@PLA-MNs (Fig. 3b). Although HA-MNs displayed higher compressive stress with the same compressive strain (greater than 5 kN) than BLM@PLA-MNs, the compressive stress of BLM@PLA-MNs could still reach almost 3 kN, which was well beyond the range of interest for applications in transdermal drug delivery systems. Additionally, we emphatically observed the strain changing tendency within 0–450 N of stress. The stress–strain curve of BLM@PLA-MNs exhibited an obvious trend variation, possibly due to the abrupt failure of BLM@PLA-MNs loaded MN tips. Excellent compressive stress of MN tips is a fundamental characteristic for penetrating the skin. As previous works have reported, HA-based MN tips possess excellent penetration capabilities [40, 41]. Furthermore, the penetrative ability of tips was demonstrated by optical photography of holes and traces created by the MN patches (loaded with rhodamine B) inserted into fresh rabbit cadaver skin (Fig. S6a). Additionally, H&E staining images of the rabbit cadaver skin after treatment with the MN patches further indicated successful penetration into the skin (Fig. S6b).

### The degradation of patches and in vitro drug release assay of BLM@PLA-MNs

After the BLM@PLA-MN patches were inserted into the skin for 10 min, the substrate was removed, and BLM@PLA MPs were released with HA MN arrays dissolving. To investigate the degradation state, BLM@PLA-MNs were applied to fresh rabbit cadaver skin to mimic a medical application setting. SEM images revealed the gradual dissolving of tips in 10 min (Fig. 3c). In addition, the morphological changes of BLM@PLA MPs within 28 days were investigated via SEM (Fig. 3d). The BLM@PLA MPs degraded gradually within 28 days in PBS and were basically completely degraded after 28 days. Therefore, the slow-release of BLM was visually confirmed. Besides, the internal morphology of MPs on the 28th day revealed a microporous structure that could load sufficient BLM.

In previous studies, PLA MPs were generally considered slow-degrading in PBS solution (pH = 7) at room temperature [42]. To assess the actual release of BLM in PBS, the UV spectrogram and standard curve of BLM were initially tested (Fig. 3e insert). The release of BLM was monitored within 28 days (Fig. 3e). As expected, once the BLM was encapsulated into PLA MPs, the release of BLM from the synthesized BLM@PLA MPs occurred continuously within 28 days, reaching a maximum (138.47 ppm, 20 mL PBS solution) at 28 days. Therefore, it could be calculated that the maximum drug release for a single MN patch was approximately 2.77 mg (138.47 mg/L × 0.02 L = 2.77 mg), which is significantly lower than the safe dosage for intralesional injection treatment of hemangiomas and vascular malformations in clinical settings [43, 44]. The initial loading amount of BLM was calculated as 164.50 ppm.

In the first 7 days, only 27.64% of the maximum BLM release amount (23.26% of the initial loading amount of BLM) was released from BLM@PLA. The releasing amount of BLM from day 7 to day 21 reached 89.09 ppm (around 64.34% of the maximum). Even from day 21 to day 28 (the last week), the amount of released BLM from BLM@PLA MPs reached 11.11 ppm, accounting for 8.02% of the maximum. The continuous release of BLM in the long-term could be attributed to the slow degradation of PLA MPs. More importantly, several explosive release processes of BLM were observed from day 2 to day 3, day 5 to day 10, and day 14 to day 21. We speculated that these results were related to the specific closed microporous structure of BLM@PLA MPs internally. More micropores were exposed to the PBS solution with the PLA degrading, leading to an explosive release of BLM.

### Biocompatibility and safety assessment of MN patches

**Fig. 4:**
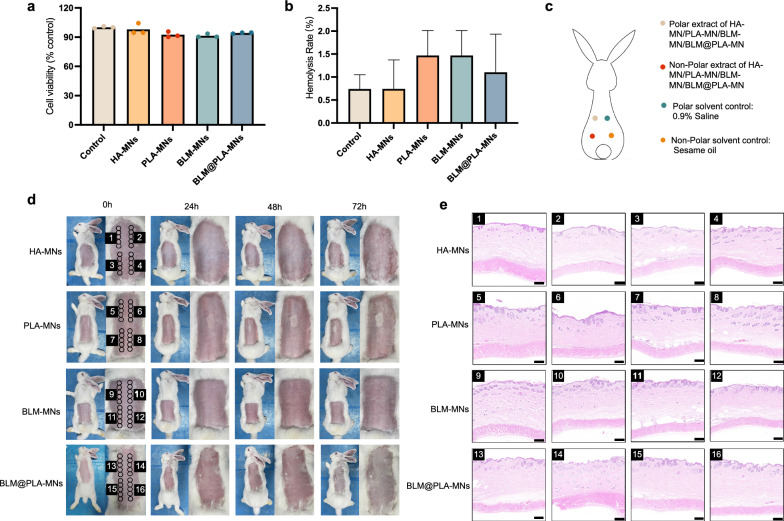
Biocompatibility and safety assessment of BLM@PLA-MNs. **a** Cytotoxicity, **b** hemolysis rate of MN patches. **c** Schematic diagram of the intradermal irritation test of MN patches. **d** Irritation and skin sensitization test of MN patches. **e** Representative histological analyses of dorsal injection points by H&E staining 72 h post-operation from MN patches treated rabbit. Scale bar: 100 μm

Ensuring biosafety is crucial for advancing the use of biomedical materials in clinical applications. Herein, the MN patches were assessed the biotoxicity by CCK8 assay. Human dermal fibroblast cells were cultivated alongside MN patches, and the cell viability was determined by measuring the OD values at 490 nm (Fig. 4a). The cell viability values for HA-MNs, PLA-MNs, BLM-MNs, and BLM@PLA-MNs were 97.85 ± 0.05%, 92.47 ± 0.02%, 91.40 ± 0.01%, and 94.27 ± 0.006%, respectively. It was worth noting that the cell viability of BLM@PLA-MNs was higher than that of BLM-MNs, indicating that BLM@PLA-MNs to some extent mitigated the impact of the drug on cells. Overall, based on ISO 10993 standards, all the prepared MN patches mentioned above can be classified as non-toxic [45]. Furthermore, the hemocompatibility of the MN patches was assessed by measuring the hemolysis rate against sheep red blood cells (Fig. 4b). It can be observed that the hemolysis rate of all MN patches on red blood cells was around 1%, significantly lower than the standard for biomedical materials (hemolysis rate < 5%) [46]. Meanwhile, the hemolysis rate of BLM@PLA-MNs was lower than that of BLM-MNs, indicating that PLA had a minor impact on the biosafety of the MN patches.

Moreover, an irritation and skin sensitization test is essential for the biological evaluation of medical devices. According to ISO 10993-10:2010 guidelines [47], the potential for MN-induced irritation reactions is assessed by injecting the MN patches extract intradermally into rabbits (Fig. 4c). The topical changes were recorded at 24 h, 48 h, and 72 h after the injections, revealing no obvious erythema, edema, or necrosis around vesicles (Fig. 4d). Histological analysis further documented microscopic features of local skin samples 72 h after the injection. Based on representative H&E-stained images, no inflammatory cells, necrotic tissue, or hematoma were detected in the subcutaneous tissues of any experimental group (Fig. 4e). In summary, the aforementioned findings suggest that the MN patches demonstrate satisfactory biocompatibility, rendering them suitable for practical medical use.

### Inhibitory effect evaluation of MN patches on hemangioma formation in vivo

**Fig. 5:**
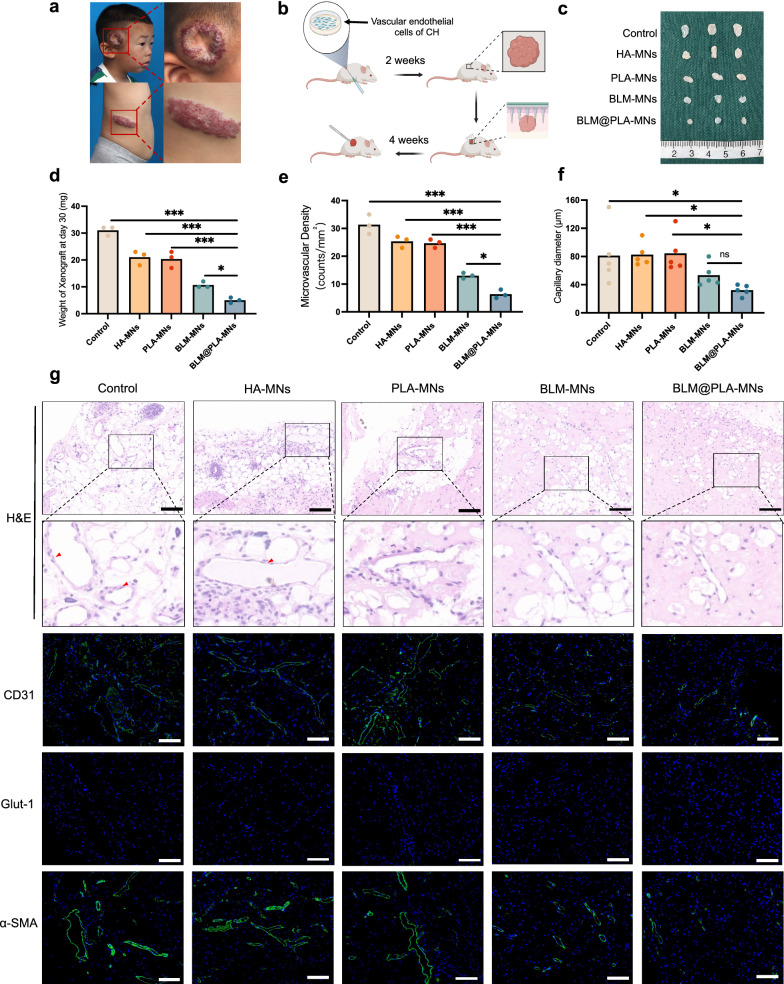
Evaluation of the inhibitory effect of MN patches in murine congenital hemangioma model. **a** Depiction of two patients of NICH (one located in the right temporal region and the other in the right waist). **b** Schematic illustration of the murine congenital hemangioma model. **c** Xenografts harvested 4 weeks after MN patch treatment. Comparison of mean weight (**d**), microvascular density (**e**), and capillary diameter (**f**) of the xenografts in each group after 4 weeks of treatment. Error bars represent mean ± SEM. n = 3 mice in each group for (**c**–**f**). **g** Representative images of H&E staining, as well as CD31, glut-1 and α-SMA levels assessed by immunofluorescence in the xenografts of each group. (n = 3 per group), *p < 0.05, **p < 0.01, ***p < 0.001. Scale bar: 100 μm

Microneedles have emerged as a promising platform for transdermal drug delivery due to enhanced permeability and reduced pain sensation [48]. In this study, we delved into the inhibitory effects of microneedle (MN) patches on hemangioma formation in vivo*.* Initially, a murine hemangioma model was established in female immunodeficiency mice by implanting vascular endothelial cells (VEC) (positive for CD31, Fig. S8b) from congenital hemangioma lesions (Fig. S8a) into Matrigel (Fig. 5a and b). The lumens in this model were positively stained for human CD31 and α-SMA, and negatively for glut-1, mirroring the expression pattern in congenital hemangioma tissues (Fig. 5g). Moreover, in the H&E sections of the control and HA-MN groups, we observed endothelial cells with a hobnailed appearance (indicated by red arrows), a common feature in congenital hemangioma tissues, reinforcing the fidelity of the model.

To evaluate the therapeutic effect of MN patches on hemangioma in vivo, we applied HA-MNs, PLA-MNs, BLM-MNs, and BLM@PLA-MNs in separate test groups. After a 10-min administration, the patches were removed, and xenografts were harvested after 30 days. Significant reductions in xenograft weight, vessel density, and capillary diameter were observed in the BLM-MNs and BLM@PLA-MNs groups (Fig. 5c–g). In comparison to BLM-MNs, BLM@PLA-MNs exhibited lower xenograft weight and microvascular density, with no significant difference in capillary diameter (Fig. 5d–f). It's noteworthy that the HA-MNs and PLA-MNs groups showed a mild decrease in weight and vascular density (Fig. 5d and e), potentially attributed to the angiogenesis inhibitory effects of HA [49]. Importantly, no pulmonary fibrosis, a common complication of bleomycin [50], was detected in the lungs of both BLM-MNs and BLM@PLA-MNs groups. Additionally, there were no significant differences in liver and kidney structures (necrosis, inflammation, steatosis) between the control group and other experimental groups (Fig. S7). These findings underscore the prolonged inhibitory effect and biosafety of BLM@PLA-MNs for hemangiomas.

However, the long-term effects of bleomycin cannot be ignored. Bleomycin can cause pulmonary toxicity, leading to conditions such as pneumonitis and pulmonary fibrosis. Although we did not observe abnormalities in the lungs, liver, and kidneys of mice during the 28-day experimental period, the long-term safety of this treatment remains uncertain. This is a limitation of our study. Future research should focus on the long-term metabolism of bleomycin in the body and its long-term side effects. Additionally, adjusting the dosage by altering the surface area of the microneedles offers a safer treatment approach for lesions of varying sizes and for patients of different ages. This remains a key focus for future research.

### BLM@PLA-MNs inhibit congenital hemangiomas via modulating the P53 pathway

**Fig. 6:**
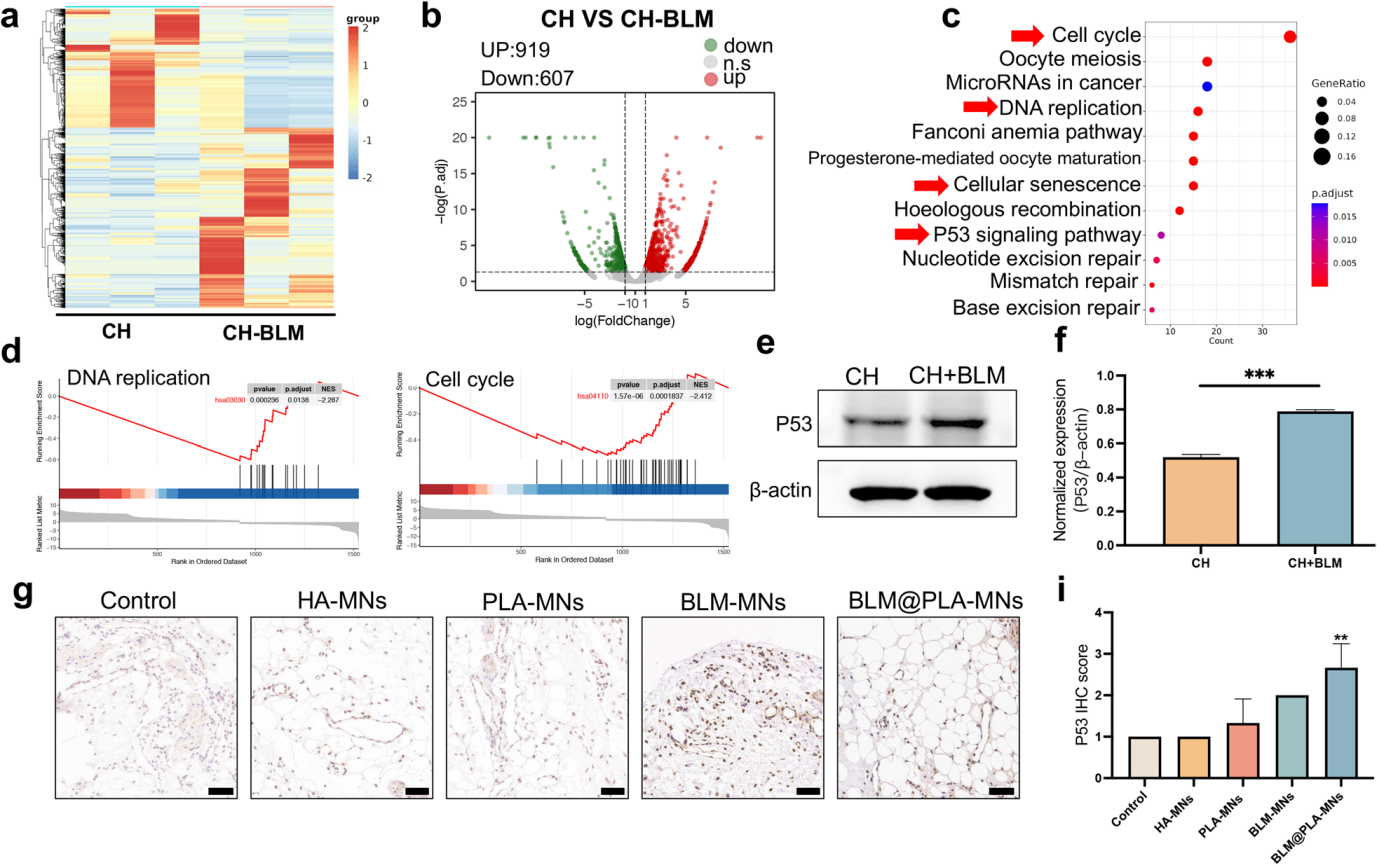
BLM@PLA-MNs inhibit congenital hemangiomas through the P53 pathway. **a** Heatmap representing color-coded expression levels of differentially expressed genes (DEGs) in CH-VEC and CH-VEC + BLM (3 patients with hemangiomas). **b** Volcano plot shows up- and down-regulated genes in CH-VEC and CH-VEC after bleomycin treatment (FC > 2, *p* < 0.05). **c** KEGG pathway enrichment analysis of DEGs. **d** Enrichment plots of select GSEA pathways enriched in CH-VEC treated with bleomycin versus control, including DNA replication and cell cycle. **e**, **f** Western blot and quantitative analysis of P53 expression in VEC of CH treated with bleomycin at concentration of 5 µg/mL for 24 h. β-actin served as the loading control. Representative images (**g**) and quantification (**i**) of P53 levels assessed by IHC in the xenografts of each group. Error bars represent mean ± SEM, n = 3 independent experiments for (**f**, **i**), *p < 0.05, **p < 0.01, ***p < 0.001. Scale bar, 50 μm

To elucidate the molecular mechanism underlying the inhibitory effects of BLM@PLA-MNs on congenital hemangiomas, RNA-sequencing (RNA-seq) was conducted on vascular endothelial cells of hemangiomas treated with bleomycin (5 μg/mL, 24 h) or PBS (control). The heatmap revealed that 919 genes were significantly up-regulated, and 607 genes were significantly down-regulated in CH treated with bleomycin compared to the control (fold change > 2, *p* value < 0.01) (Fig. 6a and b). Kyoto Encyclopedia of Genes and Genomes (KEGG) analyses indicated that multiple pathways, such as DNA replication, cell cycle, cellular senescence, and the P53 signaling pathway were enriched in bleomycin-treated group (Fig. 6c). Consistently, Gene Set Enrichment Analysis (GSEA) also revealed downregulation of DNA replication and the cell cycle (Fig. 6d). The P53 signaling pathway, known as a tumor suppressor pathway, has been shown to have connections between cell cycle arrest and DNA repair. Loss of P53 also plays a direct role in formation of the vascular malformations [51]. Moreover, bleomycin can induce DNA double-strand breaks, which, in turn, activate P53, leading to cell cycle arrest and/or DNA damage, thereby inhibiting vascular malformations [52–54]. Western blot analysis confirmed that applying bleomycin to vascular endothelial cells of CH for 24 h induced an increase in expression of P53 (P53/β-actin ratio:1.40) (Fig. 6e and f). To further confirm the bleomycin-mediated upregulation of the P53 pathway, we conducted P53 immunohistochemical staining analysis on the xenograft in the hemangioma model. In line with western blot results, BLM-MNs and BLM@PLA-MNs showed higher expression of P53, while BLM@PLA-MNs exhibited the strongest P53 expression and the smallest formation of congenital hemangioma (Fig. 6g and i). These results supported that BLM@PLA-MNs exerted an inhibitory effect on hemangioma through the upregulation of the P53 signaling pathway.

## Conclusions

In conclusion, our study presents a straightforward approach to synthesize BLM-loaded HA-based MN patches. BLM encapsulation occurred during the synthesis of PLA-MPs. Successful preparation of PLA MPs and MN patches was confirmed through SEM imaging. We thoroughly investigated the in vitro BLM release profile from PLA MPs. The safety of MN patches was comprehensively validated through cell viability assays, hemolysis ratios, and irritation and skin sensitization tests on rabbits. Moreover, we established a murine hemangioma model and demonstrated the inhibitory effects of BLM@PLA-loaded MN patches on hemangioma formation in vivo. Through RNA-Seq, western blot, and IHC, we uncovered that BLM@PLA-loaded MN patches inhibit hemangioma formation by modulating the P53 pathway. This study represents the first systematic exploration of combining PLA microspheres with microneedles to load BLM for treating hemangiomas. Our findings indicate that BLM@PLA-loaded MNs hold promise as a cost-effective and efficient treatment method for hemangiomas.

### Supplementary Information

Supplementary Material 1.

## Data Availability

The authors declare that all data supporting the results in this study are available within the paper and its supplementary information.
