# Supplementary material for: Microneedle delivery system with rapid dissolution and sustained release of bleomycin for the treatment of hemangiomas
Source: J Nanobiotechnology. 2024 Jun 25;22:372. doi: 10.1186/s12951-024-02557-7 (PMC11201781; doi:10.1186/s12951-024-02557-7)
Supplement: Supplementary file 1 — Supplementary Material 1. [file 12951_2024_2557_MOESM1_ESM.docx]

Supporting Information

**Microneedle Delivery System with Rapid Dissolution and Sustained Release of Bleomycin for the Treatment of Hemangiomas**

Bin Sun^1^, Tikai Zhang^2^, Hongrui Chen^1^, Wei Gao^1^, Jingwei Zhou^1^, Yuxi Chen^1^, Wang Ding^3^, Xiaofan Yin^3^, Jie Ren^2,*^, Chen Hua^1,*^, Xiaoxi Lin^1,*^

**Supplementary Methods:**

*Cytotoxicity evaluation*

The cell viability of human dermal fibroblasts (offered by Chinese Academy of Sciences, China) was evaluated via Cell Counting Kit-8 assay (Dojindo Molecular Technologies, Kyushu Island, Japan). Briefly, human dermal fibroblasts were seeded at 1 × 10^4^ cells per well in 1 mL Medium (Dulbecco’s Modified Eagle) for 72 h. Subsequently, the PET membrane and the prepared MN patches (measuring 1 × 1 cm²) were submerged in fibroblast cell solutions and incubated in a 24-well plate at 37°C for 72 hours. Then Cell Counting Kit-8 was carried out according to the manufacturer’s instructions. The absorbance of each well was measured at 450 nm. All the tests were repeated at least three times. The relative cell viability of the human dermal fibroblast cells was calculated based on the following formula[1]:

$$Cell viability\left( \% \right)=\frac{{OD}_{\mathrm{sample}}}{{OD}_{\mathrm{control}}}\times100\%$$

*Hemolysis assay*

Red blood cells were used aseptic defibrinated sheep blood(TX0030,Solarbio) from fresh, healthy human blood through centrifugation at 1500 rpm for 15 minutes. The resulting cells were thoroughly washed with PBS until the supernatant turned clear and transparent. Subsequently, MN patches measuring 1×1 cm² and PET membranes were immersed in a diluted blood cell dispersion (2 vol%, 2 mL per tube) to ensure full contact with the blood cells. This immersion process took place at 37 °C for 3 hours. Following immersion, 100 μL of the supernatant, obtained by centrifuging the diluted blood samples at 1500 rpm for 15 minutes, was transferred into a 96-well plate. Hemoglobin release was assessed by measuring the OD576. Negative and positive controls were established using red blood cells in PBS and 2% Triton solution, respectively. The hemolysis rate was calculated using the following formula[2]:

$$Hemolysis rate \left( \% \right)=\frac{{OD}_{\mathrm{sample}}-{OD}_{negative control}}{{OD}_{positive control}{-OD}_{negative control}} \times100\%$$

*Immunofluorescence*

Cell immunofluorescence: Cells were immobilized with 4% paraformaldehyde (PFA) for 10 minutes at room temperature, permeabilized using 0.3% Triton X-100 in PBS for 15 minutes, and obstructed with 5% bovine serum albumin (BSA) in PBS for 30 minutes at room temperature. Subsequently, cells were subjected to incubation with primary antibodies against CD31(28083-1-AP, Proteintech). Cell nuclei were stained using DAPI reagent (Abcam, ab104139). Sample analysis and image capturing were conducted utilizing a Nikon Eclipse 80i Microscope(Nikon, Japan).

Tissue immunofluorescence: The Department of Pathology at Shanghai Ninth People’s Hospital sectioned formalin-fixed, paraffin-embedded tissues from animal models. The sections underwent deparaffinization with xylene followed by hydration through a series of alcohol solutions. Antigen retrieval was conducted using a pH 9.0 antigen retrieval buffer (GT100411, GeneTech, Shanghai, China) and heated in a microwave oven for 20 minutes. After cooling to room temperature (RT), the sections subsequently blocked with 10% goat serum (dissolved in PBS) at RT for 30 min. The sections were incubated overnight with primary antibodies against CD31 (ab76533,Abcam), glut-1 (ab115730,Abcam), α-SMA (ab124964,Abcam) overnight at 4 ℃, placed in a wet box containing a little water. The sections were washed with PBS and incubated with secondary antibodies for 1 hour at RT. Next, the sections were mounted in DAPI with mounting medium and examined utilizing a Nikon Eclipse 80i Microscope(Nikon, Japan). Secondary antibodies: Goat anti rabbit FITC (Dx111-095-003,Shanghai).

*Immunohistochemistry*

Tissues were perfused with 4% PFA, stored in fixative overnight, and embedded in paraffin. For further staining with hematoxylin and eosin (H&E) and antibodies, sections of 5 μm were cut. After deparaffinization and antigen retrieval, the tissues were permeabilized with 0.3% Triton X-100, incubated with 0.3% H2O2 solution to quench endogenous peroxidase activity. Sections were blocked with goat serum and incubated with the P53 (ab32536, Abcam) at 4 ◦C overnight. After incubation with a secondary antibody, diaminobenzidine staining (DAB) (Maxim, China) was used to visualize the targets. Sectioned tissues were counterstained with haematoxylin and mounted with resin mounting medium.

Next, the slides were observed and visualized using a Nikon Eclipse Ni Microscope (Nikon, Japan).The immunohistochemical staining results were assigned mean score considering both the intensity of staining and percentage of stained cells. All sections were assessed by two researchers independently. The intensity was scored as follows:0, negative; 1, weak; 2, moderate; and 3, strong.

*Western Blot*

Proteins were extracted from vascular endothelial cells of CH treated with bleomycin or PBS 24 h using RIPA Lysis Buffer from Beyotime Institute of Biotechnology. These protein lysates were separated through sodium dodecyl sulfate-polyacrylamide gel electrophoresis (SDS-PAGE) and then transferred onto PVDF (polyvinylidene difluoride) membranes (Millipore). After blocking with 5% nonfat dry milk or bovine serum albumin (BSA) for 30 minutes, the PVDF membrane was incubated with primary antibodies overnight. Subsequently, the blots were washed with TBST and incubated with corresponding HRP-conjugated secondary antibodies from Jackson for 45 minutes. Protein visualization was achieved using the ECL Detection System from Thermo Scientific and quantification was performed using Image-Pro Plus 6.0 software. The primary antibodies used in this study were: P53 (ab32049, Abcam).

**Supplementary Data :**

**Figure S1.** SEM images and EDS (mapping) analyses of PLA and BLM@PLA MPs. The N, S elements increased obviously after PLA encapsulating BLM.

**Figure S2.** EDS (mapping) analyses of PLA and BLM@PLA MPs.

**Figure S3.** XPS spectrogram and high-resolution spectra of S2p and Cl2p of PLA and BLM@PLA.

**Figure S4.** UV-vis spectrogram of BLM before and after encapsulation of BLM.

**Figure S5.** The fluorescence microscope image of BLM@PLA-MNs. Rhodamine B (0.001 wt %) was added into the BLM@PLA-MNs in order to obtain the fluorescence microscope images.

**Figure S6**. a. The optical photography of holes and trace created by the MN tips and rhodamine B loaded MN tips inserted into fresh rabbit cadaver skin. b. H&E staining of the rabbit cadaver skin treated with the MN patches. Scale bars: 100 μm.

**Figure S7**. Representative histological analyses of liver, kidney and lung by H&E staining. Scale bars: 100 μm.

**Figure S8**. Identification of congenital hemangioma lesions and vascular endothelial cells. (a) Representative images of H&E staining, as well as CD31 and glut-1 assessed by IHC in the excised congenital hemangioma specimen. (b) immunofluorescent staining of CD31 in vascular endothelial cells (VEC) of congenital hemangioma. Scale bars: 100 μm.

**Table S1.** Clinical Characteristics of Patients

| Order | Diagnosis | Sex | Age | Focal Location | Previous treatment |
| --- | --- | --- | --- | --- | --- |
| 1 | NICH | Female | 5y | Trunk | None |
| 2 | NICH | Male | 3y | Forehead (ulceration history) | None |
| 3 | NICH | Male | 5y | Limb | None |

**Supplementary References**

[1] Chang H, Zheng M, Yu X, Than A, Seeni RZ, Kang R, et al. A Swellable Microneedle Patch to Rapidly Extract Skin Interstitial Fluid for Timely Metabolic Analysis. Adv Mater 2017;29.

[2] Yuan Y, Zhang Y. Synthesis of Imidazolium Oligomers with Planar and Stereo Cores and Their Antimicrobial Applications. ChemMedChem 2017;12:835-40.
